# Functional neuraminidase inhibitor resistance motifs in avian influenza A(H5Nx) viruses

**DOI:** 10.1016/j.antiviral.2020.104886

**Published:** 2020-10

**Authors:** Dagmara Bialy, Holly Shelton

**Affiliations:** The Pirbright Institute, Pirbright, United Kingdom

**Keywords:** Influenza viruses, Drug susceptibility, Neuraminidase inhibitors, Chickens

## Abstract

Neuraminidase inhibitors (NAIs) are antiviral agents recommended worldwide to treat or prevent influenza virus infections in humans. Past influenza virus pandemics seeded by zoonotic infection by avian influenza viruses (AIV) as well as the increasing number of human infections with AIV have shown the importance of having information about resistance to NAIs by avian NAs that could cross the species barrier. In this study we introduced four NAI resistance-associated mutations (N2 numbering) previously found in human infections into the NA of three current AIV subtypes of the H5Nx genotype that threaten the poultry industry and human health: highly pathogenic H5N8, H5N6 and H5N2. Using the established MUNANA assay we showed that a R292K substitution in H5N6 and H5N2 viruses significantly reduced susceptibility to three licenced NAIs: oseltamivir, zanamivir and peramivir. In contrast the mutations E119V, H274Y and N294S had more variable effects with NAI susceptibility being drug- and strain-specific. We measured the replicative fitness of NAI resistant H5N6 viruses and found that they replicated to comparable or significantly higher titres in primary chicken cells and in embryonated hens’ eggs as compared to wild type - despite the NA activity of the viral neuraminidase proteins being reduced. The R292K and N294S drug resistant H5N6 viruses had single amino acid substitutions in their haemagglutinin (HA): Y98F and A189T, respectively (H3 numbering) which reduced receptor binding properties possibly balancing the reduced NA activity seen. Our results demonstrate that the H5Nx viruses can support drug resistance mutations that confer reduced susceptibility to licenced NAIs and that these H5N6 viruses did not show diminished replicative fitness in avian cell cultures. Our results support the requirement for on-going surveillance of these strains in bird populations to include motifs associated with human drug resistance.

## Highlights

• A R292K residue change in NA reduces susceptibility to NA inhibitor drugs (NAIs) in H5N6 and H5N2 avian influenza viruses.
• All four mutations (E119V, H274Y, R292K and N294S) reducing susceptibility to NAIs in H5N6 viruses reduced viral NA activity.
• Reduced susceptibility to NA inhibitors in H5N6 did not attenuate virus replication efficiency in chicken cells or eggs.
• A reduction of the viral HA affinity for sialic acid was observed in H5N6 viruses with reduced NA activity.

## 1 Introduction

Wild aquatic birds are the major reservoir of avian influenza virus (AIV) worldwide. There are multiple genotypes of AIV categorised on the possession of one of 16 known surface haemagglutinin (HA) proteins and 9 neuraminidase (NA) proteins. AIVs frequently transmit from these reservoir species to other bird populations such as domestic poultry, from where they can cross additional species barriers and jump into mammalian hosts such as humans. An increase of human infections caused by different AIV subtypes in recent years (Shi et al., 2013; Gao et al., 2013; Su et al., 2017; Jiang et al., 2017; WHO, 2018a, 2019) together with evidence pointing at the avian origins of past influenza pandemics (Kawaoka et al., 1989; Webster and Laver, 1972) signals that AIV strains are a significant concern to public health.

In the absence of universal influenza virus vaccines, antivirals are important treatment and prophylaxis tools in the mitigation of AIV in humans. Whilst there has been recent progress in the development of novel influenza anti-viral drugs, such as polymerase inhibitors - represented by baloxavir marboxil approved in 2018 (O'Hanlon and Shaw, 2019), the current WHO recommended and most widely available drugs are the neuraminidase inhibitors (NAIs) (WHO, 2018b, 2018c). NAIs are small chemical compounds that bind to the active site and surrounding framework residues of the viral NA enzyme. NAIs prevent the NA from cleaving sialic acid and thus reduce influenza virus replication by impeding egress of virions from host cells and their dissemination to new target cells. Currently there are only two world-wide licenced NAIs: oseltamivir (OSE) and zanamivir (ZAN), with peramivir (PER) licensed and used in Japan, South Korea, EU and USA and laninamivir licensed and used in Japan only (Shie and Fang, 2019).

Antiviral drug resistance of influenza virus is known to develop following treatment of humans with the NAI drugs for both human influenza strains (H1N1 and H3N2) and AIVs (H7N9 and H5N1) (Gubareva et al., 2001; Kiso et al., 2004; Le et al., 2005; Marjuki et al., 2015). Indeed, the passage of influenza virus in the presence of NAI drugs has been demonstrated for multiple subtypes of influenza A virus (Choi et al., 2018; Song et al., 2015). There are known motifs that confer reduced susceptibility to the NAI drugs: E119V, H274Y, R292K and N294S (amino acid numbering refers to the N2 subtype), amongst others but the level of reduced susceptibility and which drug they affect are not universal amongst the influenza A virus NAs or even NA subtype.

The error prone polymerase of the influenza virus results in natural diversity that may, albeit rarely, in the absence of a drug selection pressure result in natural NAI resistance by AIVs. An analysis performed by Govorkova et al. (2013), examined the prevalence of NAI resistance associated mutations in H5N1 AIV sequences deposited in the GenBank database from 2002 to 2012. This analysis revealed that 0.8% of H5N1 AIV sequences (N = 1716) contained a resistance motif (defined as E119A, H274Y, or N294S) (Govorkova et al., 2013). In addition, a study by Boltz et al. (2010),found that several H5N1 viruses (3/29) from chickens and ducks in Laos contained motifs which conferred reduced sensitivity to oseltamivir. A meta-analysis done by Orozovic et al. (2011), showed that out of 5490 avian NA protein sequences on the NCBI database, 132 (2.3%) had at least one mutation associated with NAI resistance in human N1 and N2 subtypes. However, the publication did not examine whether the sequences conferred functional resistance to NAIs.

Understanding the signatures that confer resistance to antiviral drugs for currently circulating strains of virus is therefore important information. Determination of whether resistance motifs alter the fitness of the viruses that possess them is also of importance - it is likely that viruses prevalent in migratory birds could be transmitted to domesticated poultry and thus subsequently find their way across the avian – mammalian host barrier. The fitness of influenza A viruses relies in part on the functional balance between the two surface glycoproteins: HA, which binds to cell surface sialic acid to enable attachment and entry of the virus to cells; and NA, which cleaves sialic acid to allow egress and dissemination of the new virions to additional target cells or a new host organism. The 9 NA subtypes found in AIV isolates are phylogenetically split into two groups: Group 1 comprising N1, N4, N5 and N8, and Group 2 including N2, N3, N6, N7 and N9 (McAuley et al., 2019).

In the past five years a lineage of highly pathogenic H5 viruses, known as the H5Nx viruses, have caused multiple worldwide outbreaks in poultry. The H5Nx viruses all possess the same clade 2.3.4.4 H5 HA protein of the A/goose/Guangdong/1/1996 highly pathogenic H5N1 virus origin (Antigua et al., 2019). Reassortment events between the widespread H5N1 and viruses circulating in waterfowl and domestic poultry have resulted in H5N2, H5N3, H5N6 and H5N8 responsible for significant outbreaks and causing economic and animal welfare concerns for the global poultry industry. In addition, H5N6 has sporadically crossed over to humans (24 confirmed cases) and caused severe disease (Jiang et al., 2017; WHO, 2018). The prevalence of the H5Nx strains in birds and the frequency of poultry outbreaks makes these viruses of significant concern for human health. There is little current knowledge about the functionality of known NAI resistance associated mutations in the H5Nx viruses.

In this study we introduced four NAI resistance-associated mutations (E119V, H274Y, R292K, N294S) into three recent H5Nx viruses containing different avian NA subtypes: N2, N6 and N8. To maintain optimal HA/NA balance during replication in avian substrate all recombinant viruses carried H5 HA with multibasic cleavage site of the original strain. We determined the susceptibility of these viruses to three licensed NAIs: OSE, ZAN and PER. Furthermore, for the H5N6 subtype (which has caused infection of humans already), we assessed virus replication efficiency of selected drug-resistant strains in primary chicken kidney cells (cKC) and embryonated hen's eggs, and compared the NA activity and HA receptor binding to viruses carrying a wild type (wt) NA.

## 2 Materials and methods

### 2.1 Cells, viruses and plasmids

Madin-Darby Canine Kidney (MDCK; ATCC) and human embryonic kidney cells (HEK 293T; ATCC) were maintained as described previously (James et al., 2016). Primary chicken kidney cells (cKC) were prepared in house and maintained as described elsewhere (James et al., 2016; Hennion and Hill, 2015).

The following NA and HA genes were synthesised by GeneArt (Invitrogen) and cloned into a pHW2000 vector (Hoffmann et al., 2000):A/chicken/Jiangxi/02.05 YGYXG023-P/2015 (H5N6) (**GISAID accession nos. EPI661558 and EPI661559**); A/scarlet_ibis/Germany/AR44-L01279/2015 (**GISAID accession nos. EPI624533 and EPI624535**) (H5N8); A/goose/Taiwan/01031/2015 (H5N2) (**GenBank accession nos. KU646887 and KU646885**. Single nucleotide changes in NA were introduced using QuikChange™ site-directed mutagenesis protocol (Stratagene). For NA activity assay H5N6 NAs with C-terminal FLAG tag were subcloned into pCDNA3.1(+) vector (ThermoFisher). All recombinant viruses were generated using an eight plasmid reverse genetics system (Hoffmann et al., 2000) with 6 internal genes (PB2, PB1, PA, NP, M, NS) originated from A/Puerto Rico/8/1934 (H1N1; further referred to as PR8) and matching HA/NA from a chosen strain (Table 1). Viruses were propagated in embryonated hen's eggs as described previously (James et al., 2016). The HA activity of produced virus stocks (haemagglutinating units, HAU/ml) was measured by haemagglutination assay (Hirst, 1942) with chicken red blood cells (cRBC), and the infectious titres (plaque forming units, PFU/ml) were determined by plaque assay on MDCK cells (Table 2). The introduced NA mutations were maintained upon rescue and passage in eggs with no additional mutations in the NA gene appearing as confirmed by sequencing.

**Table 1:** Panel of generated recombinant influenza strains (2:6) HxNx: PR8 and analysis of NAI resistance-associated markers in H5Nx NA sequences deposited on the GISAID database.

Group	Subtype	HA/NA	Strain	Prevalence of NAI resistance-associated mutations in AIVs 2013–2018 (E119V/D/Q, H274Y, R292K, N294S)
N2-like	**N2**	**H5N2** HPAIV clade 2.3.4.4	A/goose/Taiwan/01031/2015	E119 V/D/Q - None H274R – 1/462 (0.2%) R292K – None N294S - None
	**N6**	**H5N6** HPAIV clade 2.3.4.4	A/chicken/Jiangxi/02.05 YGYXG023-P/2015	E119D – 26/1356 (1.9%) aE119D - 1/23 (4.3%) H274Y – 1/1356 (0.1%) R292K - None N294S – 3/1356 (0.2%)
N1-like	**N8**	**H5N8** HPAIV clade 2.3.4.4	A/scarlet ibis/Germany/Ar44-L01279/2015	E119Q - 1/967 (0.1%) H274R – None R292K – None N294S – None

a – human AIV isolates.

**Table 2:** Infectious [PFU/ml] and haemagglutinating [HAU/ml] titres of recombinant viruses carrying wt or mutated NA.

Subtype	Strain	NA	Infectious titre^a^ [PFU/ml]	Haemagglutinating titre^a^ [HAU/ml]
**N6**	**H5N6** A/chicken/Jiangxi/02.05 YGYXG023-P/2015	Wt	3.6 × 10^7^	2560
		E119V	6.1 × 10^7^	2560
		H274Y	4.5 × 10^6^	640
		R292K	2.7 × 10^7^	1280
		N294S	1.0 × 10^8^	5120
**N8**	**H5N8** A/scarlet ibis/Germany/Ar44-L01279/2015	Wt	2.9 × 10^5^	1280
		E119V	1.4 × 10^7^	2560
		H274Y	3.9 × 10^4^	160
		R292K	2.4 × 10^5^	Undetectable
		N294S	1.9 × 10^7^	2560
**N2**	**H5N2** A/goose/Taiwan/01031/2015	Wt	2.4 × 10^7^	5120
		E119V	7.0 × 10^7^	5120
		H274Y	1.6 × 10^7^	2560
		R292K	6.8 × 10^6^	1920
		N294S	2.3 × 10^7^	5120

a Infectious titre and HA titre following rescue and one passage in embryonated hen's eggs.

All viruses carrying H5 HA with multibasic cleavage site and/or NA modified to increase the resistance to licensed NAIs were handled in the SAPO4/BSL3 containment facility of The Pirbright Institute by trained and authorised personnel.

### 2.2 Validation of FLAG-NA expression and NA activity assay on live cells

HEK 293T cells were transfected with 1 μg of H5N6 FLAG-NA-expressing plasmid for 24 h and harvested in either 1 × Laemmli buffer (Laemmli, 1970) for validation of NA expression, or the isotonic buffer (32.5 μM MES, 4 mM CaCl_2_, 1% FCS; pH 6.5) for live-cell NA activity assay. Protein lysates were analysed by Western blot with α-FLAG (Sigma-Aldrich) and α-GAPDH (Invitrogen) antibodies, and the band intensities were quantified using ImageJ software (Schneider et al., 2012). NA activities were measured using the fluorogenic substrate MUNANA (2'-[4-Methylumbelliferyl]-alpha-D-N-acetylneuraminic acid; Biosynth), as described previously (World Health OrganizationWHO Global Influenza Surveillance Network, 2011), normalised to protein amount and expressed as percentage of wt.

### 2.3 NA inhibition assay

Fluorescence-based NA inhibition assays were performed using the fluorogenic substrate MUNANA, live viruses and NA inhibitors: oseltamivir carboxylate (OSE; MedChem Express), zanamivir (ZAN; Sigma-Aldrich) and peramivir (PER; SelleckChem) as described elsewhere (World Health OrganizationWHO Global Influenza Surveillance Network, 2011). Virus drug resistance was classified according to the WHO recommendations based on the fold increase in IC_50_ compared to the susceptible reference strain (wt NA): normal inhibition (NI) < 10-fold; reduced inhibition (RI) 10 to 100-fold; highly reduced inhibition (HRI) > 100-fold. (Anonymous, 2012).

### 2.4 Virus titration by plaque assay

Infectious virus from harvested allantoic fluid or cell supernatant was titrated by plaque assay on MDCK cells as described before (James et al., 2016), with modified overlay medium: 0.6% agarose (Oxoid) or 0.6–0.8% Avicel® Microcrystalline Cellulose and Sodium Carboxymethylcellulose (FMC BioPolymer).

### 2.5 Plaque reduction assay

Plaque reduction assay (PRA) on MDCK cells was performed as described elsewhere (Hayden et al., 1980), with modifications. Equal volumes of virus inoculum containing 30–80 PFU per well were added to confluent cells in 12-well plates. After 1 h adsorption at 37 °C virus inoculum was removed and cells were cultured with 0.6% Avicel overlay supplemented with NAIs: OSE, ZAN or PER. The final drug concentrations in medium ranged in 4-fold dilutions from 0.04 nM to 10 μM; each condition in duplicate. After 72 h virus plaques were visualised by fixation with 0.1% crystal violet containing 20% methanol and the diameters of at least 10 plaques per condition were measured using ImageJ software (Schneider et al., 2012). The EC_50_ represents half maximal effective concentration of drug leading to 50% reduction in plaque size as compared to no drug. The data was analysed in Excel (MS Office) and the EC_50_ values were calculated using Graph Pad Prism software (GraphPad Prism version 8.00, www.graphpad.com).

### 2.6 Multi-cycle virus growth curves *in vitro* and *in ovo*

For growth curve *in vitro*, cKC cells in 6-well plates were inoculated with virus at an MOI of 0.001 (4000 PFU/ml), each virus in triplicate as described previously (James et al., 2016). The supernatant was collected at 8, 24, 48 and 72 h post infection. To determine the inhibitory effect of NAI on virus growth in cKC, cells infected with wild type virus were cultured in the presence of 10 μM OSE. All samples were titrated by plaque assay. The growth curves *in vitro* were repeated at least twice.

For growth curve *in ovo* 10-day old embryonated hen's eggs were inoculated into the allantoic cavity with 100 PFU (1000 PFU/ml) of virus in PBS supplemented with 1% penicillin/streptomycin and 0.35% BSA as described previously (James et al., 2016). Five eggs per each virus and time point were sampled at 10, 24 or 48 h post inoculation. To measure the inhibitory effect of NAI on virus growth *in ovo*, H5N6 wt virus was pre-incubated with 100 μM PER for 45 min prior to inoculating into eggs as described elsewhere (Tare and Pawar, 2015). The dose of PER was adjusted based on the embryo size as well as the volume of allantoic fluid per egg to increase chances of even distribution and tissue penetration. To monitor any adverse effects of drug on embryo survival, three eggs were inoculated with 100 μM PER in virus diluent. All samples were titrated by plaque assay.

### 2.7 Receptor binding avidity assay

Receptor binding avidity assay of recombinant viruses was performed as described elsewhere (Hensley et al., 2009), with modifications. Briefly, cRBCs were pre-treated with increasing concentrations (1–500 U/ml) of recombinant NA from *Clostridium perfringens* (New England Biolabs). 4 HAU of each virus was added to sialidase-treated 1% [v/v] cRBCs solution and incubated for 1 h at 4 °C. The highest enzyme concentration allowing for full virus haemagglutination was recorded [U/ml] and expressed as a relative receptor binding avidity.

### 2.8 Sanger sequencing of viral RNA

Viral RNA (vRNA) was extracted and purified from allantoic fluid using QIAmp Viral RNA kit (Qiagen) and cDNA was synthesised using Verso cDNA reverse transcription kit (ThermoFisher Scientific) and influenza universal primers (Hoffmann et al., 2001). The HA and NA segments were amplified using subtype-specific primers. Nucleotide sequences were determined with BrilliantDye™v3.1 Terminal Cycle Sequencing Kit (Nimagen), strain-specific primers and Applied Biosystems 3730 DNA Analyzer. All primers are available upon request.

### 2.9 Cell viability assay

MDCK and cKC were cultured for 72 h in the presence or absence of NAI drugs, each condition in triplicate. Relative cell viability was quantified based on the presence of ATP as an indicator of metabolically active cells using CellTiter-Glo® Luminescent Assay (Promega) according to manufacturer's protocol.

### 2.10 Statistical analyses

Statistical analyses were performed in Minitab® Statistical Software (LLC, USA; www.minitab.com) using general linear model with post hoc Dunnett's test (NA activity, growth curve in cKC, receptor binding avidity assay) or Kruskal-Wallis and Mann-Whitney nonparametric tests (growth curve in eggs).

## 3 Results

### 3.1 Prevalence of human influenza virus NAI resistance-associated substitutions in AIV

**Fig. 1:**
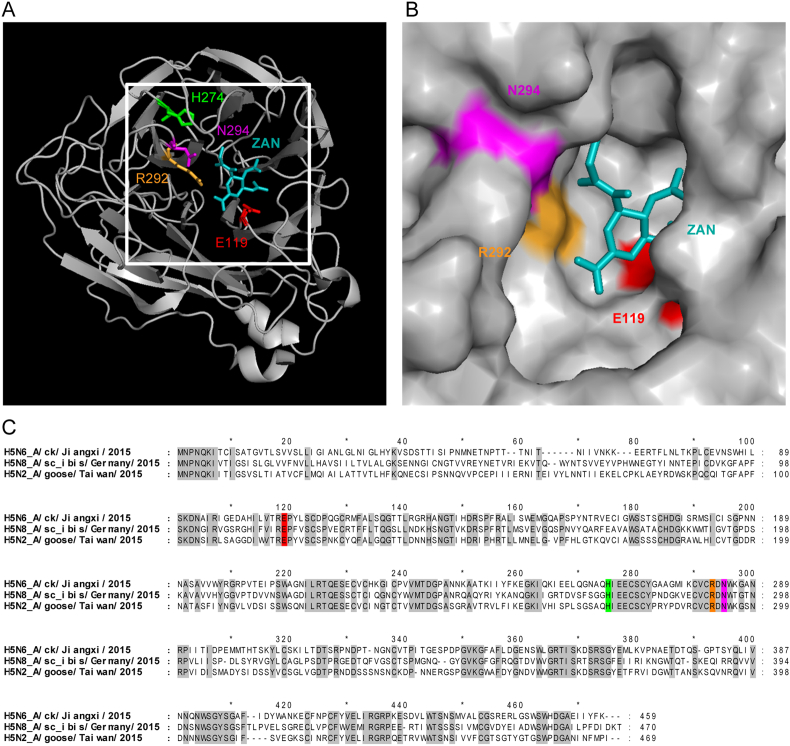
**Location of potential neuraminidase inhibitor (NAI) resistance mutations in NA catalytic site.** Four residues critical for binding of NAI drugs – three framework (E119, H274, N294) and one catalytic (R292) are highlighted in colours. **(A)** Crystal structure of H11N6 A/duck/England/1/1956 NA complexed with zanamivir (ZAN; blue) (PDB 2CML). **(B)** Surface area of ZAN binding pocket within the NA active site. **(C)** Protein sequence alignment of H5N6, H5N8 and H5N2 NAs with conserved residues shown in grey.

The four most common NAI reduced susceptibility associated mutations in human influenza strains (H1N1 and H3N2) are E119V, H274Y, R292K and N294S (N2 NA subtype numbering) (Gubareva et al., 1996, 2001; Kiso et al., 2004; Abed et al., 2006; McKimm-Breschkin et al., 1998; Tai et al., 1998; Wang et al., 2002; Sleeman et al., 2013; Zhang et al., 2014). Two of these mutations, R292K and E119V, have also been reported in human isolated H7N9 viruses following NAI drug treatment (Marjuki et al., 2015). The R292 residue is one of the catalytic residues of NA and thus critical for the enzymatic function. The other three are framework residues (E119, H274 and N294) stabilizing the structure of the active site of NA (Fig. 1). We assessed the prevalence of these four mutations in the NA proteins of recent H5Nx viruses. We included avian isolated H5N2, H5N6 and H5N8 and human isolated H5N6 AIV sequences deposited in GISAID database between January 1, 2013, when the first bird infections with H5N6 were reported, and August 2019. We identified H274Y substitution in 1 viral sequence (0.1%) and N294S in 3 sequences (0.2%) of the 1356 avian H5N6 isolates (Table 1). None of the avian or human H5N6 carried E119V signature, however 4.3% of human and 1.9% of avian strains contained Aspartic acid (D) at this position, a mutation recently reported to confer pan-resistance to available NAIs in pH1N1 (Baek et al., 2015). In the avian H5N8 NA sequences a single sequence with a mutation from E119 to a Glutamine (Q) and in the avian H5N2 sequences a single sequence with an Arginine (R) at position 274 instead of a Histidine (H) were observed.

### 3.2 Susceptibility profile of H5Nx viruses to licenced neuraminidase inhibitors (NAIs)

**Fig. 2:**
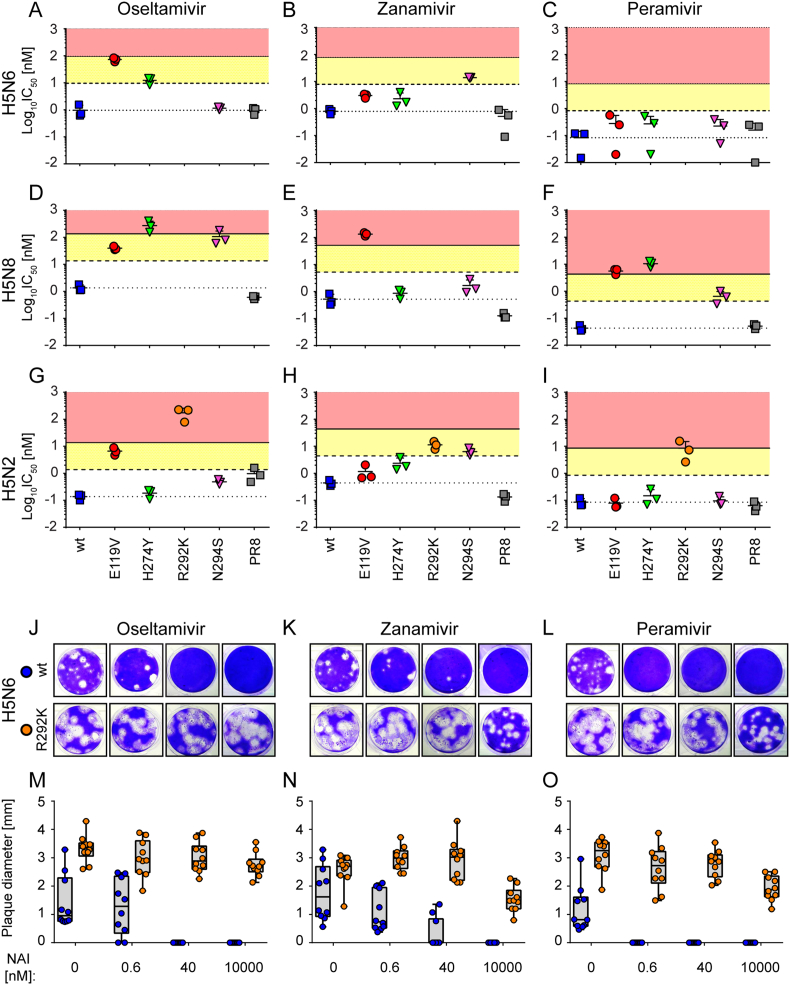
**Neuraminidase inhibitor (NAI) susceptibility profile of H5N6, H5N8 and H5N2 influenza viruses. (A**–**I)** MUNANA assay. The IC_50_ values for three H5Nx viruses: H5N6 (A–C), H5N8 (D–F) and H5N2 (G–I) were determined by fluorescent NA inhibition assay. Each panel of viruses (R292K only for H5N2) alongside with the PR8 strain were tested for their susceptibility to OSE (left column), ZAN (middle column) and PER (right column). Short horizontal line with error bar indicates the mean of log_10_IC_50_ [nM] ± SD of three independent experiments; each data point represents a single experiment. The area between the dotted and dashed lines on each graph represents the normal inhibition (NI; <10-fold increase in IC_50_ over corresponding wt), the yellow-shaded area - reduced inhibition (RI; 10 - 100-fold increase in IC_50_) and the red-shaded area – highly reduced inhibition (HRI; 100<-fold increase in IC_50_). **(J**–**O)** Susceptibility profile of H5N6 carrying R292K NA substitution to NAIs as determined by plaque reduction assay. MDCK cells were infected with H5N6 viruses bearing either wt (blue) or R292K (orange) NA variants. The cells were cultured with increasing NAI concentrations (0–10 μM): OSE (left column), ZAN (middle column) or PER (right column). The plaques were developed after 72 h of incubation (J–L) and the plaque sizes were measured for at least 10 plaques per condition (M–O). Box and whiskers graphs in M-O represent the median plaque diameter with minimum and maximum values [mm]; each data point represents a single plaque.

We next determined the susceptibility of the generated viruses to three NAI drugs: oseltamivir (OSE), zanamivir (ZAN) and peramivir (PER) using the MUNANA fluorescence-based NA inhibition assay to generate IC_50_ values. IC_50_ is the concentration of drug that will reduce the NA activity by 50% from no drug (Fig. 2 and Table 3). With the exception of H274Y mutation in human H1N1 viruses (HRI to OSE and PER) there is currently not enough data to correlate these categories of IC_50_ change to clinical implication for an infected host (Kawai et al., 2009; Memoli et al., 2010). It was not possible to determine the IC_50_ value by MUNANA assay for the H5N6 and H5N8 R292K mutant as the fluorescent signal generated using MUNANA did not reach the recommended threshold for signal-to-background ratio. PR8 was used as a reference strain in each experiment to monitor the inter-assay variability, and its NAI susceptibility profile was consistent with the one reported by others (Yen et al., 2007).

**Table 3:** The impact of introduced mutations on virus susceptibility to licensed NAI drugs in different HA/NA backgrounds.

NA/Subtype	wt	E119V	H274Y	R292K	N294S
OSELTAMIVIR IC_50_ ± SD [nM]					
**H5N6** n=3^a^	0.96 ± 0.51^b^ 2.27 [PRA]^c^	72.6 ± 12 **(75)**^d^	12.35 ± 3.5 **(13)**	[>10000; PRA]^e^	1.14 ± 0.14
**H5N8** n=3	1.35 ± 0.39	39.8 ± 6.38 **(30)**	271.97 ± 125.76 **(202)**	n.d.^f^	106.8 ± 65.1 **(79)**
**H5N2** n=3	0.14 ± 0.03 0.2 [PRA]	6.6 ± 2.09 **(48)**	0.18 ± 0.06	173.7 ± 82.67 **(1268)** 866.8 **(4365)** [HRI; PRA]	0.48 ± 0.1
**ZANAMIVIR IC**_**50**_**± SD [nM]**					
**H5N6** n=3	0.79 ± 0.17 2.23 [PRA]	3.02 ± 0.52	2.31 ± 1.5	4482 **(2008)** [HRI; PRA]	13.9 ± 0.75 **(18)**
**H5N8** n=3	0.52 ± 0.27	132.87 ± 18.79 **(255)**	0.86 ± 0.3	n.d .	1.67 ± 1.02
**H5N2** n=3	0.44 ± 0.11 0.5 [PRA]	1.16 ± 0.77	2.34 ± 1.33	11.3 ± 3.6 **(26)** 14.2 **(28)** [RI; PRA]	6.33 ± 2.05 **(14)**
**PERAMIVIR IC**_**50**_**± SD [nM]**					
**H5N6** n=3	0.08 ± 0.06 0.15 [PRA]	0.28 ± 0.28	0.27 ± 0.25	2580 **(17200)** [HRI; PRA]	0.22 ± 0.17
**H5N8** n=3	0.04 ± 0.01	5.6 ± 1.27 **(130)**	10.49 ± 2.66 **(244)**	n.d.	0.65 ± 0.33 **(15)**
**H5N2** n=3	0.09 ± 0.03 0.06 [PRA]	0.08 ± 0.04	0.15 ± 0.1	8.46 ± 6.52 **(100)** 5.00 **(84)** [RI; PRA]	0.1 ± 0.04

a Number of independent MUNANA assays.

b Data represent mean of n independent experiments ± standard deviation (SD).

c EC_50_ determined by Plaque Reduction Assay.

d Numbers in parentheses indicate fold increase in IC_50_ over the corresponding wt NA; shown only for reduced inhibition (RI; 10 - 100-fold) or highly reduced inhibition (HRI; >100-fold).

e Drug susceptibility determined by Plaque Reduction Assay, although dose response curve not converged (no precise EC_50_ could be retrieved).

f Not determined.

The OSE IC_50_ values for the E119V mutation in all three viruses demonstrated RI, the H274Y mutation resulted in RI for the H5N6 NA and HRI for the H5N8 NA, and N294S mutation showed RI only in H5N8 background (Fig. 2A, D & G). The R292K mutation in H5N2 resulted in HRI for OSE (Fig. 2G). For H5N6 wt and R292K viruses we used plaque reduction assay (PRA) and determined the EC_50_ value for each drug by measuring reduction in plaque size as response to the NAIs. The highest concentration of OSE (10 μM) completely abrogated plaque formation of wt H5N6, whereas no reduction in plaque size was observed for H5N6_R292K (Fig. 2J & M) which suggests HRI of R292K virus to OSE. Addition of NAI drugs to MDCKs did not affect the cell viability (Fig. 5A).

The results of MUNANA assay with ZAN showed that E119V mutation conferred HRI only in H5N8 virus, N294S mutation led to RI in H5N6 and H5N2 backgrounds and H274Y substitution did not affect the susceptibility to ZAN in any of the H5Nx viruses (Fig. 2B, E & H). The H5N2_R292K virus demonstrated RI to ZAN (Fig. 2H), and in the plaque reduction assay the H5N6_R292K virus showed a 40% reduction in plaque size (10 μM of ZAN) compared to no drug, giving the phenotype of HRI when compared to wt H5N6 (Fig. 2K and N).

The IC_50_ measurements made using the MUNANA assay for PER with the panel of H5N6 mutants did not show any alteration in susceptibility to the drug, and for the H5N2 panel only the R292K showed RI for PER (Fig. 2C and I). H5N8_E119V and H5N8_H274Y viruses both displayed HRI for PER whereas H5N8_N294S showed a more modest RI to the drug (Fig. 2F). In the plaque reduction assay addition of 10 μM PER reduced the plaque size of the H5N6_R292K by approximately 40%, and thus resulted in HRI when compared to the 100% reduction in wt H5N6 virus (Figure 2L & O). All the values for the drug susceptibility of all the wt and mutant viruses for the H5Nx panels are summarised in Table 3.

### 3.3 Mutations conferring reduced susceptibility to NAIs reduce the enzymatic activity of the H5N6 NA

**Fig. 3:**
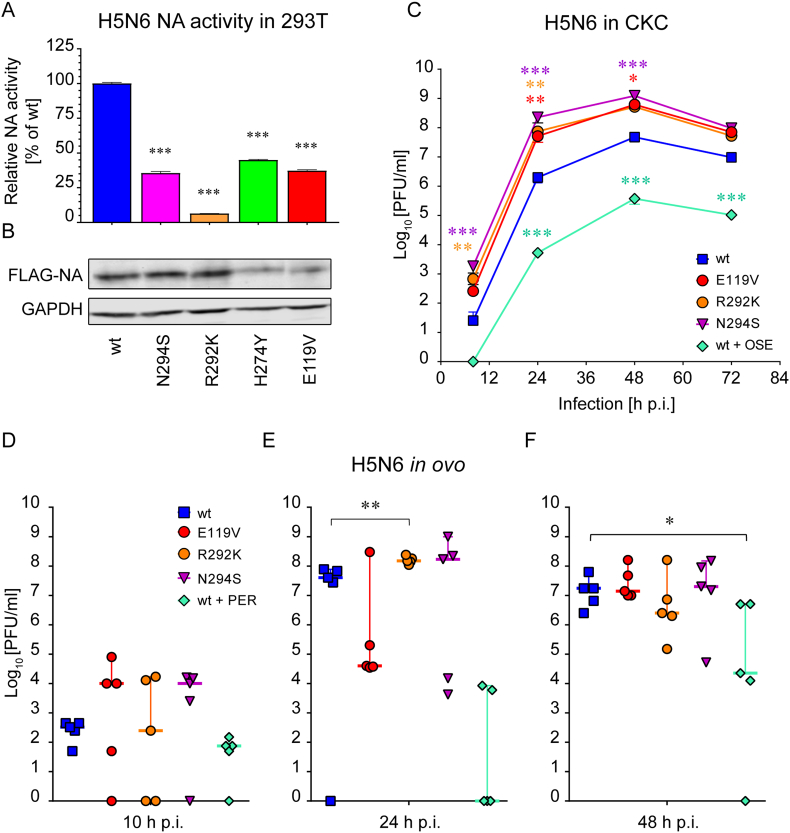
**NA activity profile and growth kinetics of NAI-resistant H5N6 viruses *in vitro* and *in ovo*. (A)** NA activities of H5N6 NAs. FLAG-tagged NAs (wt, E119V, H274Y, R292K and N294S) were expressed in HEK 293T cells, the NA activities were determined by MUNANA assay and normalised to protein expression levels. Data represent mean of 3 independent experiments ± SD (***p < 0.001). **(B)** Western blot analysis of H5N6 FLAG-NA expression in HEK 293T cells. Band intensities were quantified using ImageJ software and levels of protein expression were normalised to GAPDH. **(C)** Growth curve *in vitro*. cKC were infected with H5N6 NAI-resistant viruses as well as the corresponding wt at an MOI of 0.001. The supernatants were collected at the indicated time points and titrated by plaque assay. Graphs shown are representative of 2 independent experiments; each data point represents mean of 3 replicates ± SD (*p < 0.05; **p < 0.01; ***p < 0.001). **(D**–**F)** Growth curve in ovo. 10 day-old SPF embryonated hen's eggs were inoculated with H5N6 viruses at 100 PFU/egg. Allantoic fluid samples were collected at the indicated time points and titrated. Each data point represents a single egg; short horizontal line indicates the median with 95% CI, n = 5 (*p < 0.05; **p < 0.01). All virus-infected embryos were viable at 10 and 24 h p.i. and reached the terminal end point by 48 h p.i.

To investigate the impact of introduced mutations on NA activity we performed MUNANA assay on live HEK 293T cells expressing recombinant H5N6 NAs at the cell surface. The enzyme activity was normalised to NA protein levels as determined by Western blot and displayed as the percentage of wt (Fig. 3 A & B). The E119V and H274Y NAs were expressed at lower levels compared to the wt NA (64% and 69% of wt, respectively; Fig. 3B), and those differences were considered while normalising the NA activities to protein expression. The results from 3 independent experiments showed that all four mutations significantly impaired the enzymatic activity when compared to wt NA (p < 0.001). The R292K mutation reduced NA activity to 6% of the wt NA, N294S to 35%, E119V to 37% and H274Y to 45% (Fig. 3A).

### 3.4 Chicken *in vitro* and *in ovo* replication kinetics of NAI-resistant H5N6 viruses

**Fig. 4:**
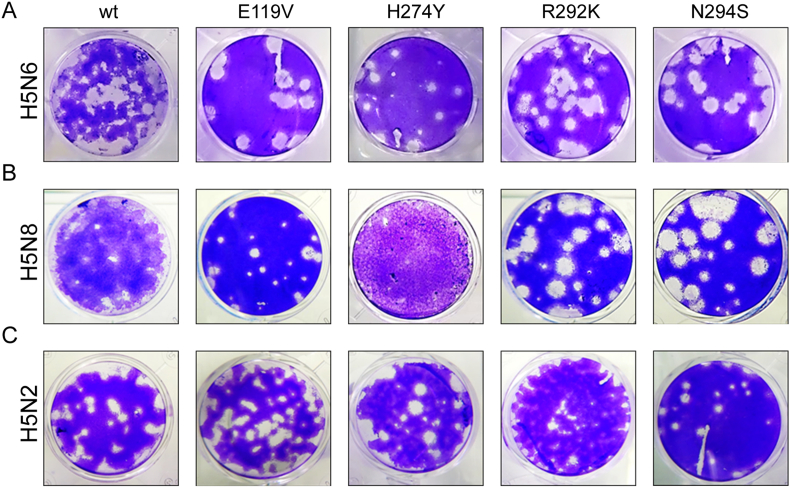
**Plaque morphology of wt and NAI-resistant viruses on MDCK cells.** MDCK cells were infected with a panel of H5N6 (A), H5N8 (B) and H5N2 (C) viruses and cultured for 72 h with agarose (A and C) or Avicel® overlay medium (B). Viral plaques were visualised with crystal violet staining.

To understand whether the introduced mutations affected virus replication we used primary chicken kidney cells (cKC) and assessed the replicative capacity of the H5N6 AIV subtype bearing NAI resistance-associated markers. Three H5N6 mutant viruses, E119V, N294S and R292K showed reduced susceptibility to at least one drug (Fig. 2 and Table 3), and despite impaired NA activity (Fig. 3A) grew to comparable titres as the wt H5N6 virus during passage one in hens’ eggs (Table 2) and were able to spread from cell to cell in an MDCK cell monolayer (Fig. 4A).

**Fig. 5:**
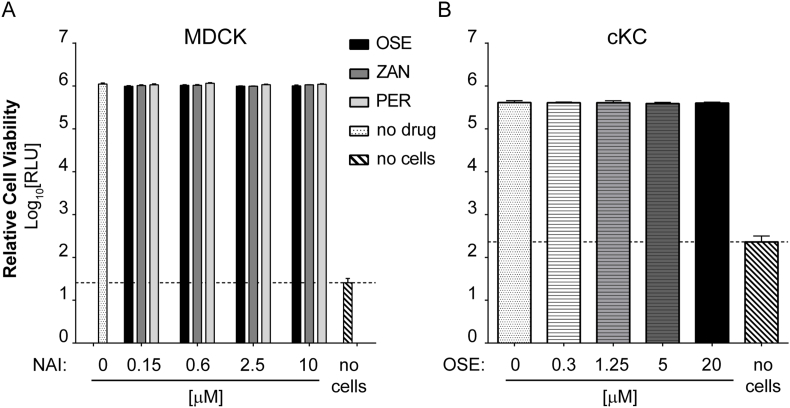
**Cell viability of MDCK and cKC cultured in the presence of NAIs.** MDCK (A) or cKC (B) cells were seeded in 96-well black-walled plates and cultured in the presence of NAI drugs for 72 h: (A) 0.15, 0.6, 2.5 or 10 μM of OSE (black bars), ZAN (grey bars) or PER (light grey bars); (B) 0.3, 1.25, 5 or 20 μM OSE, followed by cell viability assessment using luminescent assay (RLU). Untreated cells are shown as white dotted bar, and dashed line on the graph indicates background levels (no cells) also displayed by the white bar with diagonal stripes.

We inoculated primary cKC at an MOI of 0.001 PFU/cell with each virus and collected the supernatants at the indicated time points (Fig. 3C). All three NAI-resistant H5N6 viruses as well as the wt showed the peak of viral replication at 48 h post infection (Fig. 3C). All three mutant viruses replicated significantly faster when compared to wt H5N6 (N294S at 8, 24 and 48 h p.i, p < 0.001; R292K at 8 and 24 h p.i, p < 0.01; E119V at 24 h p.i., p < 0.01 and 48 h p.i., p < 0.05). The addition of 10 μM OSE to cKC infected with wt H5N6 significantly reduced the titres of cell-free virus when compared to cells with no drug (24, 48 and 72 h p.i., p < 0.001) demonstrating that NA activity is required for replication of AIV in primary chicken cells in culture (Fig. 3C). The concentrations of up to 20 μM OSE did not alter the cKC cell viability (Fig. 5B).

To verify the effects we observed in cKCs we inoculated 10-day old embryonated hen's eggs with drug-resistant H5N6 viruses, sampled the eggs at indicated time points and determined the median titres of infectious virus (Fig. 3 D – F). At 10 and 24 h p.i. all virus-infected embryos remained alive as determined by egg candling. At 10 h p.i. the median titres of H5N6_E119V and N294S viruses reached ~1 × 10^4^ PFU/ml, which was ~2-log more than wt H5N6 and R292K, however the differences were non-significant (Fig. 3D). At 24 h p.i. wt H5N6 together with two NAI-resistant mutants, R292K and N294S replicated to their highest titres between 4 × 10^7^–1.5 × 10^8^ PFU/ml (Fig. 3E), and the numbers of infectious particles dropped at 48 h p.i. (Fig. 3F), when all the embryos reached their terminal end points as indicated by extensive haemolysis of supporting blood vessels. In contrast to other viruses, E119V showed a small increase in titre between 10 and 24 h p.i. (from 1 to 4 × 10^4^ PFU/ml) and reached the maximum of 1.4 × 10^7^ PFU/ml only at 48 h p.i. The differences in virus titres for all three mutants (E119V, R292K and N294S) were non-significant (p > 0.05) as compared to wt H5N6 with exception of R292K at 24 hpi which replicated to significantly higher titres (p < 0.01).

Pre-incubation of wt H5N6 with 100 μM PER significantly reduced viral titres at 48 h p.i. when compared to eggs inoculated with virus only (p < 0.05), but it was not enough to completely abrogate virus production (Fig. 3D–F). The virus growth of NAI-resistant H5N6 variants in eggs was not significantly impaired compared to the corresponding wt, thus based on the results of our *in vitro* and *in ovo* fitness assessment we concluded that the NAI-resistant H5N6 viruses are able to replicate at least as efficiently as the wild type in tested models.

### 3.5 Compensatory mutations in HA of NAI-resistant H5N6 and their impact on receptor binding avidity

**Fig. 6:**
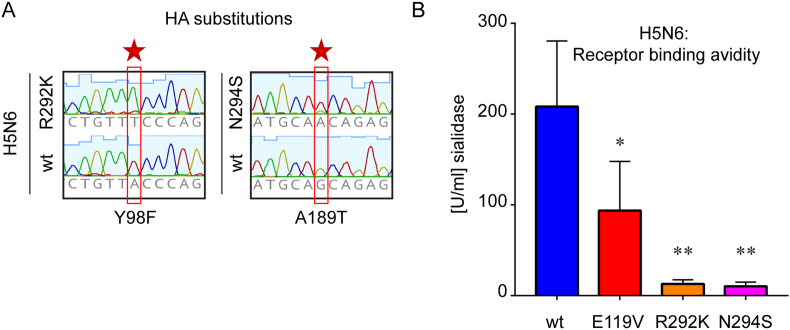
**Concurrent changes in HA of NAI-resistant H5N6 viruses and their impact on receptor binding avidity.** (A) Amino acid substitutions found in HA genes of H5N6_R292K and N294S viruses. HA segments were amplified from viral RNA and analysed following Sanger sequencing. Changes on nucleotide level are indicated with a red frame and a star; amino acid substitutions are shown below (H3 numbering). (B) Receptor binding avidity of H5N6 viruses. Relative receptor binding avidity was determined with 4 HAU of each virus on partially desialylated cRBCs pre-treated with recombinant NA from *C. perfringens*. The Y-axis shows mean ± SD of maximal NA concentration allowing for full haemagglutination of each virus (U/ml); n = 3 (*p < 0.05; **p < 0.01).

Concurrent mutations in HA can re-balance changes affecting NA activity therefore we analysed the HA sequences of NAI-resistant H5N6 viruses propagated in eggs. Two H5N6 mutants acquired single amino acid substitutions in their HA following rescue and stock preparation in eggs: Y98F (H3 numbering) in the R292K NA mutant and A189T in N294S NA mutant virus (Fig. 6A). Both mutations have previously been shown to alter receptor binding affinity in human strains (Martin et al., 1998; Wu et al., 2012). We used partly desialylated cRBCs to explore the impact of Y98F and A189T mutations in H5N6 HA on receptor binding avidity (Fig. 6B). Both HA mutants showed significantly reduced receptor binding avidity as compared to wt: the Y98F HA mutation (R292K virus) led to 94% reduction and the A189T HA mutation (N294S virus) to 95% reduction in binding avidity (both p < 0.01). Surprisingly, the NA E119V mutant, despite lack of compensatory mutations in HA also exhibited decreased receptor binding avidity by ~55% (p < 0.05).

## 4 Discussion

In this study we investigated whether known resistance mutations that occur in human isolated IAV strains (E119V, H274Y, R292K and N294S) function to reduce the susceptibility to NAI drugs in the highly pathogenic H5Nx viruses (H5N2, H5N6 and H5N8) that have been responsible for large scale poultry outbreaks in recent years. In contrast to previous reports investigating functional NAI resistance signatures in AIV carrying 7 genes (including HA) from laboratory strain PR8 (H1N1) (Choi et al., 2018; Song et al., 2015) we used recombinant viruses with matching HA/NA set of the tested strain and 6 internal genes from PR8 (2:6). Our approach provided an advantage of looking at the contribution of functional HA/NA balance to virus replication efficiency in avian substrate, as it was previously reported that such balance is required to maintain good viral fitness and mis-matching HA of certain receptor binding affinity with NA of different enzymatic activities could perturb this fine equilibrium and affect virus replicative capacity (Gaymard et al., 2016). Although the use of internal genes from PR8 may be seen as a limitation of this study, it eliminates additional source of variation such as contribution of polymerase complex to replication efficiency, and offers increased safety for the staff handling zoonotic strains carrying HPAI H5 HA and NAI-associated resistance mutations in NA. As has been demonstrated in other published studies we found no universal mutation that reduced the sensitivity of all three NA subtypes to all NAI drugs, (OSE, ZAN and PER) (Fig. 2, Table 3). The caveat with this being that the R292K mutation reduced the NA activity to such low levels that for the H5N6 and H5N8 viruses we were unable to read reliable fluorescence signals and thus determine the sensitivity level to tested compounds. In the H5N2 background the R292K mutation reduced sensitivity to all three drugs tested as determined by MUNANA assay (Fig. 2, Table 3) and confirmed by plaque reduction assay (Table 3), and a similar impact on the drug-susceptibility profile was observed for the H5N6 virus using the plaque reduction assay (Fig. 2, Table 3). The R292K mutation alters one of the three residues in the active site of the NA enzyme responsible for direct binding to the sialic acid substrate and sialic acid analogue compounds. Published work has demonstrated reduced multi-drug effects conferred by the 292K mutation in the H3N2 and H4N6 strains (Choi et al., 2018; Abed et al., 2006). We observed that the E119V mutation reduced the inhibition to OSE for all three H5Nx virus strains (Fig. 2, Table 3). Mutation at position E119 has been observed to cause reduced inhibition to OSE in both group 1 (H5N1) and group 2 NA subtypes (H3N2 and H7N9) previously (Kiso et al., 2004; Marjuki et al., 2015; Abed et al., 2006; Baek et al., 2015), although it is reported most commonly in group 2 NA subtypes. The H274Y mutation in all three H5Nx virus backgrounds did not demonstrate any reduction in inhibition for ZAN (Fig. 2, Table 3). The largest effect of the H274Y mutation on susceptibility to OSE and PER was observed with the N8 NA, the only group 1 NA in our panel which agrees with the published literature that the H274Y mutation causes reduced inhibition against OSE and PER for group 1 NAs (Le et al., 2005; Abed et al., 2006). Moreover, E119V and H274Y mutations in N8 displayed similar NAI susceptibility profile to the one reported in respective pH1N1 mutants – with E119V showing RI to all three NAIs, and H274Y to OSE and PER (Pizzorno et al., 2011).

The four mutations: E119V, H274Y, R292K and N294S did therefore confer variable reduced drug susceptibility to the NA proteins of highly pathogenic H5N2, H5N6 and H5N8. Some mutations such as E119D/Q, H274Y/R or N294S, albeit rare in avian isolated H5Nx viruses, were found to have naturally arisen (Table 1) and we would predict that the strains we identified in the database of the H5N6 and H5N8 subtype with an alternative E119 residue in the NA coding region and the H5N6 H274Y mutation would result in these viruses having functionally reduced susceptibility to OSE. Since these mutations appeared in avian isolated strains, we assume these are spontaneously arising mutations in the absence of a drug pressure unlike the human isolated avian H5Nx sequences which will have been recovered from patients hospitalised and subjected to anti-viral treatment with NAI drugs.

Usage of the oral drug OSE has increased dramatically for the treatment and prophylaxis of seasonal human influenza infection in Japan and the USA since the swine flu pandemic of 2009 (Zaraket and Saito, 2016). The active form of drug is not efficiently absorbed by the human body and a substantial amount (>80%) is excreted by the body in urine and thus into waste water following oral administration (He et al., 1999). There are multiple reports of significant OSE levels in European rivers and in water and surrounding sewage works in Japan where up to 10% of the population use the drug during the influenza season (Ghosh et al., 2010). One potential consequence of OSE in waste water and rivers is the contact that water fowl, the main reservoir of AIV strains, have with the drug. In experimental scenarios mallard ducks that consumed OSE in the water whilst infected by AIV of a H1N1, H5N2, H7N9 or H6N2 strain, did develop and shed virus that was phenotypically characterised as resistant to OSE, suggesting that this scenario could also play out in nature (Achenbach and Bowen, 2013; Gillman et al., 2013, 2015a, 2015b).

We also know from previous experience that unregulated use of human anti-viral drugs in farmed animals can occur by poultry farmers under pressure from a constant avian influenza threat. In the late 1990s there is evidence of the unregulated use of the anti-viral compound amantadine in farmed poultry in China (Cyranoski, 2005). Subsequently, the re-emerged highly pathogenic H5N1 virus in poultry in 2003 was amantadine resistant. Today a high proportion of the circulating avian influenza viruses possess mutations such as L26I or S31N, which confer resistance to amantadine (Govorkova et al., 2013; Hurt et al., 2007). Substantial and continuous outbreaks of AIV in farmed poultry species in recent years which include the H7N9 outbreak in China, the widespread H9N2 and H5N1 endemicity problem in Asia and North Africa, and the recent global H5Nx viruses provide a similar economic and social crisis that could tempt unregulated use of the OSE drug in poultry. Gilead first patented OSE and granted an exclusive licence to Roche for production in 1996 who branded the drug Tamiflu (Lew et al., 2000). However, since 2006 an increasing number of generic versions have come to the market in various countries including India (Cipla produces Antiflu) and the USA (Natco Pharma produces generic capsules of different strengths) (Lobo, 2016). These generic versions have cut the cost of the Tamiflu drug by two-thirds.

Known mutations that confer reduced susceptibility to the NAI drugs are in the active site of NA and the associated framework residues that maintain the active site structure. We and others have shown that the NA mutations which result in reduced susceptibility to the NAI drugs often also have a reduced NA activity compared to the wt NA viruses and this can impact viral replication fitness, particularly *in vivo* where mucus provides a barrier to infection and transmission (Govorkova, 2013). Therefore, the presence in avian species of a virus with reduced susceptibility to NAI drugs may in reality be of little consequence when in competition with viruses that are fitter in the absence of a drug pressure. To assess the replication efficiency of NAI-resistant H5N6 viruses we used avian primary cells and embryonated hen's eggs. Although MDCK cells are widely used in the field for virus titration and propagation, they are of mammalian origin (canine) and our viruses carry avian HA and NA with preference for avian cells. In addition there is evidence that influenza viruses are able to replicate in continuously growing (immortalised) cell lines in a NA-independent manner thus primary cells and embryonated eggs provided the optimal model (Roberts et al., 2015; Mori et al., 2011). Our results for H5N6 viruses in primary chicken kidney cells and embryonated hen's eggs, where NA activity is an important determinant of fitness (as demonstrated by the addition of an NAI to the wild type sensitive virus resulting in reduced viral replication), showed that the mutant viruses had an equal or improved replication capacity (Fig. 3). Prior to the 2009 pandemic the seasonal H1N1 human virus that was circulating contained a H274Y mutation in the NA gene that conferred reduced susceptibility to OSE. The H274Y mutation alone resulted in reduced viral fitness but additional NA mutations that occurred concurrently enabled this mutation to be supported as they increased the NA activity of the viruses (Duan et al., 2014). It has also been observed that concurrent mutation of the HA gene, reducing the affinity for sialic acid is another mechanism by which influenza A viruses may support the carriage of NAI reduced susceptibility which leads to NA activity reduction (Blick et al., 1998; Hurt et al., 2009). We observed in two of our H5N6 viruses with reduced sensitivity to NAI drugs that HA changes were also present following rescue and initial passage in embryonated hens' eggs. Investigation of the two viruses carrying these mutations, Y98F (R292K NA mutant) and A189T (N294S NA mutant), showed a reduced binding to sialic acid as was reported in the literature for these mutations in H3N2 and H1N1, respectively (Wu et al., 2012; Abed et al., 2002). Concurrent mutations in the surface glycoproteins to those that induce reduced susceptibility to the NAI drugs may also confer fitness on influenza viruses by altering the epitopes that are targeted by neutralising antibodies thus enabling immune escape (Ilyushina et al., 2019). This is of particular concern if NAI drugs are given in an unregulated way to farmed poultry in the arena of endemic outbreaks where vaccines may also be being utilised.

In summary, our study has shown that the NAI resistance-associated mutations commonly found in human influenza A strains also reduce the susceptibility to those drugs in recently emerged avian H5Nx viruses. These mutations can reduce viral NA activity and prompt the virus to acquire additional changes to allow for efficient replication in host cells, e.g. by changing the receptor binding avidity of viral HA. Changes in HA can directly affect virus host tropism or facilitate immune escape which may impact zoonotic potential of the H5Nx AIV viruses.

## Funding information

This work described herein was funded by 10.13039/501100000268BBSRC grant BB/M023362/1 and BBRSC Institute Strategic Programme Grants BBS/E/I/00007037 and BBS/E/I/00007039. The funders had no role in study design, data collection, data interpretation, or the decision to submit the work for publication.

## Author contributions

The work was conceptualised by HS and DB. Experimental work was executed by DB. The manuscript was written and edited by DB and HS.

## Declaration of competing interest

None declared.
